# Clinical, demographic, and genetic risk factors of treatment‐attributed suicidality in >10,000 Australian adults taking antidepressants

**DOI:** 10.1002/ajmg.b.32913

**Published:** 2022-07-14

**Authors:** Adrian I. Campos, Enda M. Byrne, Frank Iorfino, Chiara Fabbri, Ian B. Hickie, Cathryn M. Lewis, Naomi R. Wray, Sarah E. Medland, Miguel E. Rentería, Nicholas G. Martin

**Affiliations:** ^1^ Department of Genetics and Computational Biology QIMR Berghofer Medical Research Institute Brisbane Queensland Australia; ^2^ School of Biomedical Sciences, Faculty of Medicine The University of Queensland Brisbane Queensland Australia; ^3^ Institute for Molecular Bioscience The University of Queensland Brisbane Queensland Australia; ^4^ Child Health Research Centre The University of Queensland Brisbane Queensland Australia; ^5^ Brain and Mind Centre The University of Sydney Camperdown New South Wales Australia; ^6^ Social, Genetic and Developmental Psychiatry Centre Institute of Psychiatry, Psychology and Neuroscience, King's College London London UK; ^7^ Department of Biomedical and Neuromotor Sciences University of Bologna Bologna Italy; ^8^ Queensland Brain Institute The University of Queensland Brisbane Queensland Australia

**Keywords:** antidepressants, genetics, suicide

## Abstract

Emergence of suicidal symptoms has been reported as a potential antidepressant adverse drug reaction. Identifying risk factors associated could increase our understanding of this phenomenon and stratify individuals at higher risk. Logistic regressions were used to identify risk factors of self‐reported treatment‐attributed suicidal ideation (TASI). We then employed classifiers to test the predictive ability of the variables identified. A TASI GWAS, as well as SNP‐based heritability estimation, were performed. GWAS replication was sought from an independent study. Significant associations were found for age and comorbid conditions, including bipolar and personality disorders. Participants reporting TASI from one antidepressant were more likely to report TASI from other antidepressants. No genetic loci associated with TAS I (*p* < 5e‐8) were identified. Of 32 independent variants with suggestive association (*p* < 1e‐5), 27 lead SNPs were available in a replication dataset from the GENDEP study. Only one variant showed a consistent effect and nominal association in the independent replication sample. Classifiers were able to stratify non‐TASI from TASI participants (AUC = 0.77) and those reporting treatment‐attributed suicide attempts (AUC = 0.85). The pattern of TASI co‐occurrence across participants suggest nonspecific factors underlying its etiology. These findings provide insights into the underpinnings of TASI and serve as a proof‐of‐concept of the use of classifiers for risk stratification.

## 1 INTRODUCTION

Depression is a highly prevalent complex mental disorder (Brody, Pratt, & Hughes, 2018; Hasin et al., 2018; Kilkkinen et al., 2007) with heterogeneous clinical manifestations and multiple physical (Himmerich et al., 2008; Kang et al., 2015) and psychological comorbidities (Hasin et al., 2018). It is one of the leading causes of disability worldwide and a major contributor to the overall global burden of disease (Lopez & Murray, 1998). Antidepressants are commonly prescribed medications (Hall et al., 2003; Sharma, Guski, Freund, & Gøtzsche, 2016) which have been proven effective at treating depression in randomized controlled trials (Bridge et al., 2007; Cipriani et al., 2018; Khan, Warner, & Brown, 2000). Nonetheless, treatment response to antidepressants is heterogeneous. Reports suggest that effectiveness and side effects vary from one individual to another and from one antidepressant to another.

The emergence or worsening of suicidal symptoms after antidepressants is perhaps one of the most controversial potential side effects. A series of meta‐analyses on the emergence of suicidal ideation in adolescents and children (Hammad, Laughren, & Racoosin, 2006) motivated a warning by the US Food and Drugs Administration. There have been several attempts to elucidate whether treatment‐emergent suicide ideation (TESI) is a true side effect of antidepressants when compared to placebo and other therapies (Bridge et al., 2007; Khan, Khan, Kolts, & Brown, 2003; Perlis et al., 2007; Plöderl & Pascal, 2019; Pompili et al., 2010; Rucci et al., 2011; Sharma et al., 2016). Furthermore, the effectiveness of the FDA warning has been challenged (Friedman, 2014; Stone, 2014). In 2010, a study using a definition of *definitive suicidal behavior* on a sample of 14,911 subjects from 57 RCTs identified an association between paroxetine treatment and TESI in MDD patients (Kraus et al., 2010). Claims on antidepressant efficacy and trial implementation have been published, arguing that antidepressant side‐effects might not outweigh their benefits (Fountoulakis & Möller, 2011; Fournier et al., 2010; Khin, Chen, Yang, Yang, & Laughren, 2011; Kirsch, 2014; Rabkin et al., 1986). Nonetheless, more recent studies have concluded that antidepressant benefits outweigh suicidality increased risk (Dragioti et al., 2019).

Given that TESI only seems to occur in a subset of individuals (Healy, Bechthold, & Tolias, 2014), a plausible hypothesis is that a distinct underlying etiology could explain it. Thus, some studies have attempted to identify potential clinical (Kraus et al., 2010; McCall et al., 2010; Zisook et al., 2009) and genetic correlates of TESI (Laje et al., 2007; Perroud et al., 2009; Perroud et al., 2011). Nonetheless, these studies have been performed on relatively small samples or been focused on a reduced number of antidepressants. These promising, albeit likely underpowered approaches, raise the possibility of enabling clinicians to make an informed choice on whether to prescribe antidepressants on a case by case basis.

Here we assess the prevalence, clinical, demographic and genetic risk factors of self‐reported treatment attributed suicidal ideation (TASI), as a proxy for TESI, in the Australian Genetics of Depression Study (AGDS) cohort. AGDS comprises a large sample of Australian adults treated with antidepressants and participated through several online questionnaires and donation of a spit sample for DNA profiling. We hypothesized that the underpinnings of TASI would overlap with those of broad sense suicidality, including genetic, neurobiological, and environmental components. As such, therapies with some evidence of protective effects against suicidality, such as lithium and ketamine (De Berardis et al., 2018), might be effective adjuvants or combinations for those identified at high risk of TASI. The present study aimed to identify clinical, demographic, and genetic risk factors for TASI and assess their predictive ability to inform precision psychiatry.

## 2 METHODS

### 2.1 Participants

The AGDS was recruited through two streams. Invitations to participate in the *Australian Genetics of Depression Study* were mailed out on behalf of the investigators by the Australian Government Department of Human Services to individuals with antidepressant prescriptions according to the nationwide Pharmaceutical Benefits Scheme database. In parallel, a nation‐wide recruitment campaign was conducted using both conventional and electronic social media. Potential participants filled out detailed online questionnaires comprising clinical and sociodemographic variables used to ascertain eligibility. The DHS did not share any information about the participants invited to participate. All participants provided informed consent before participating in the study. The QIMR Berghofer Human Research Ethics Committee approved this study and all questionnaires used. Inclusion criteria involved the provision of informed consent and willingness to supply a saliva sample for later DNA extraction (*n* = 18,217). A detailed description of the sample characteristics and the recruitment strategy is available elsewhere (Byrne et al., 2020).

### 2.2 Comorbidity and TASI assessment

For each antidepressant they had taken, participants were asked to complete a standardized instrument based on the *Antidepressants Efficacy and Side Effects Questionnaire* (Li, Tian, Seabrook, Drevets, & Narayan, 2016), which we used to gather information on a variety of antidepressant side‐effects including suicidal ideation or attempt. Data were collected as a binary outcome (yes/no) for each patient and antidepressant. Participants were defined as presenting TASI if they self‐reported experiencing suicidal ideation as a direct consequence or side‐effect from at least one antidepressant. Participants who reported suicidality before antidepressant treatment or missing data on the onset of suicidality or antidepressant usage were excluded from the analyses (N_excluded_ = 7,804). Moreover, participants were also asked whether they had ever been diagnosed with specific comorbidities and provided information on their worst depressive episode characteristics.

### 2.3 Statistical analyses

TASI risk factor odds ratios (ORs) were calculated using logistic regression modeling, with TASI as the outcome variable. Multivariate logistic regressions were performed, including all variables of interest plus age and sex as covariates unless otherwise stated. Coefficient significance was estimated using Wald‐tests after Bonferroni correction. A power analysis (see Supplementary Methods) indicated that the total sample size should enable us to detect OR differences as small as ~0.1 with 80% power. TASI correlations across different antidepressants were estimated using the tetrachoric correlations (ρ), which assume a bivariate liability threshold model and are considered an appropriate model for estimating correlations between binary variables. Logistic regressions and genomic‐restricted maximum likelihood were used to perform the GWAS and the SNP‐based heritability (see supplementary methods).

### 2.4 TASI prediction

We were interested in testing the predictive capability of the predictive variables identified; thus, we decided to try different supervised learning algorithms for predicting TASI. Five different classifiers (Naïve Bayes, decision tree, AdaBoost, random forests, and logistic regression; see Supplementary Methods) were built to test the predictive power of the observed risk factors. These are simple classifiers with different underlying assumptions. A detailed description is available in supplementary methods. The outcome (or random variable) to be predicted was defined to be TASI, and the selected features were: age, sex, marital status, comorbidities, history of antidepressant usage and depressive symptoms. Given the lack of robust genetic results, the classifiers were trained on the demographic and clinical variables (Tables S1, S2, S3) but no genetic factors. The dataset was randomly divided into training (66.6% of the sample) and testing (33.3% of the sample) subsets to avoid overfitting. The different models' predictive power was assessed by computing the area under the receiver operative curve and the precision recall curve and compared against the performance of a random guess model. The best‐fitting methods were subjected to a validation analysis consisting of 100 replications randomly splitting the data into training and testing subsets and computing the receiver operating characteristic (ROC) curve and precision recall (PR) curves. The PR curve is of importance in this study as the ROC curve can be affected by an imbalance in the number of cases and controls.

### 2.5 Validation by TASA probability distributions

The best models were further assessed on their ability to stratify treatment attributed suicide attempts (TASA). Briefly, 100 subjects from each category (TASA, TASI and controls) were randomly selected and held out from the training to be used as a testing set. The model was trained to predict TASI on the remaining subjects and used to compute the mean TASI probability (or decision function) across the three groups. This procedure was repeated 1,000 times; the distribution for mean TASI probabilities was depicted using boxplots and compared using *t* tests.

### 2.6 Out of sample prediction

To further validate the predictive value of our model, we performed TASI prediction on the Australian Genetics of Bipolar Study (AGBS) as of 17/09/20. The total sample size was 4,910 participants. After applying the same exclusion criteria, the sample consisted of 1,652 participants, of which 323 were deemed as TASI cases. To ensure no sample overlap, participants self‐reporting a diagnosis of bipolar disorder were removed from the AGDS training dataset. We tested for out of sample prediction in both directions: training in AGDS and predicting TASI in AGBS and training in ABGS and predicting in AGDS.

## 3 RESULTS

### 3.1 Sample TASI prevalence

**FIGURE 1:**
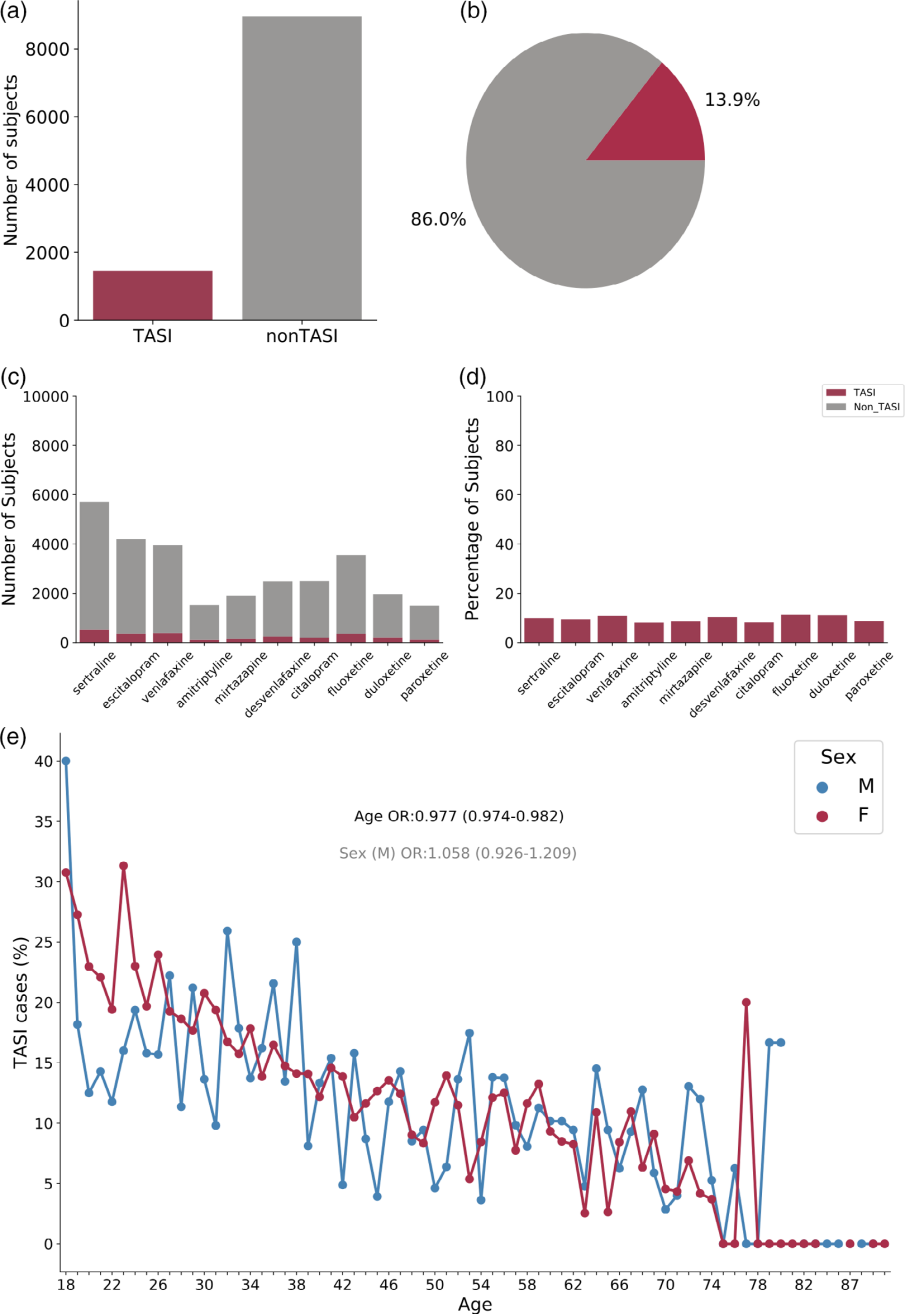
TASI prevalence is independent of antidepressants prescribed. Number (a,c) and percentage (b,d) of subjects reporting TASI across all antidepressants (a,b) and stratified by antidepressant used (c,d). Note the similar prevalence (~13%) regardless of antidepressant. (e) TASI prevalence across ages and stratified by sex in our sample. Odds ratios are shown within the figure

Between the different antidepressants, the mean TASI prevalence was ~9% in the AGDS sample. All participants had taken at least one antidepressant, and ~ 14% of all subjects (1,455 unique cases; estimate regardless of antidepressant taken) met the criteria for TASI, that is, self‐reported suicidal thoughts, self‐reported attribution to the antidepressant treatment and no suicidality prior to antidepressant intake (Figure 1a,b). Despite variation and overlap in the number of participants reporting intake of different antidepressants, the ratio of TASI to *controls* (non‐TASI individuals) was similar across antidepressants (Figure 1c,d). To assess whether one of the antidepressants explained all of the variance in TASI, we fitted a logistic regression jointly comparing TASI across the different antidepressants. This analysis showed all of them were associated with an increase in TASI risk (Figure S1). We estimated that, for each year increase in age, there was a ~ 2.3% reduction in TASI risk (OR: 0.977 [0.974–0.982]). Furthermore, our results suggested an increased risk for divorced or separated participants compared to those who are married (Table S1), and no significant differences between TASI across male and female participants (OR: 1.058 [0.926–1.209]; Figure 1e).

### 3.2 TASI risk is independent of the antidepressant type

**FIGURE 2:**
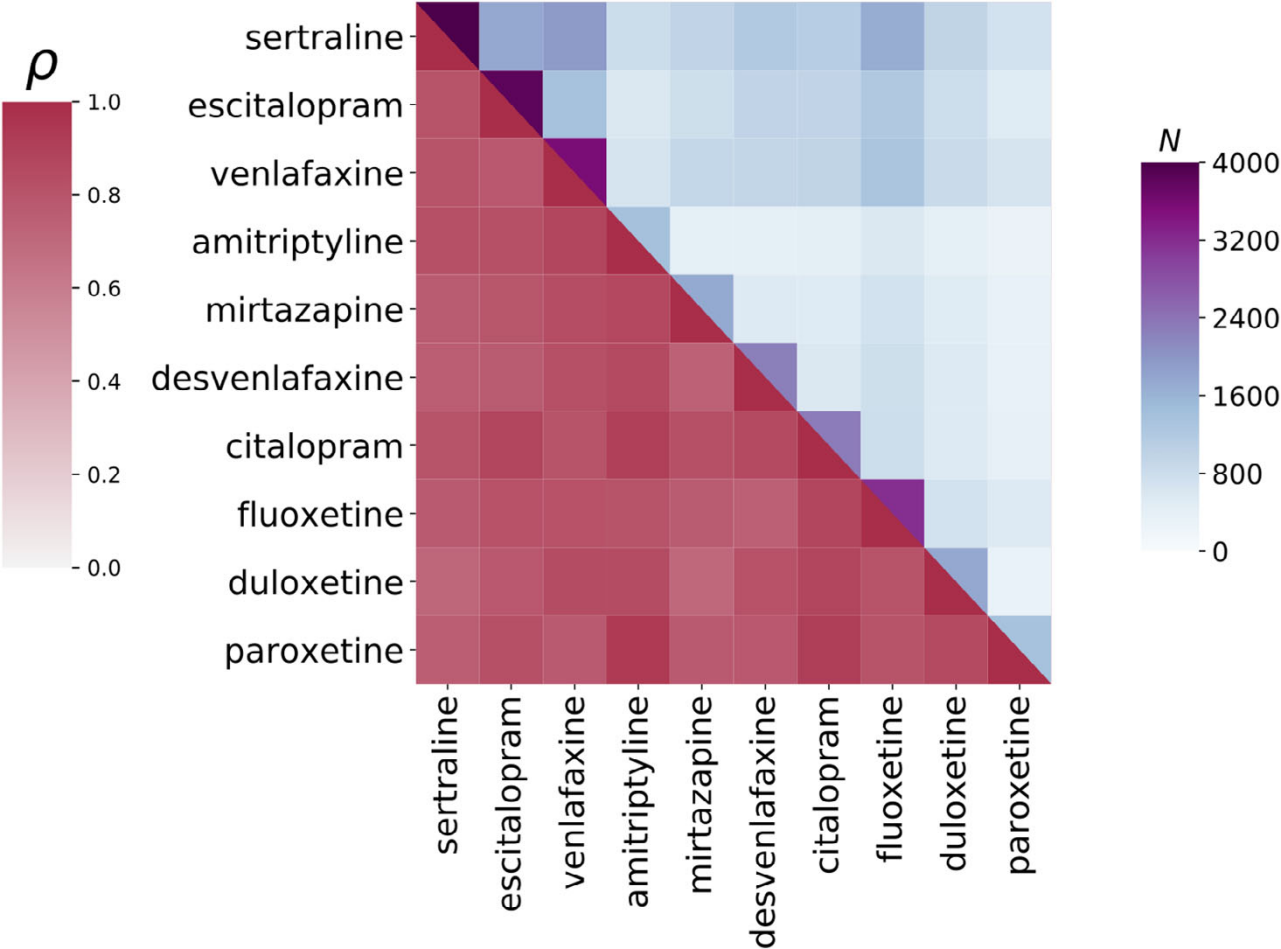
TASI correlations suggest a common underlying mechanism for all tested antidepressants. Within‐subject antidepressant TASI correlations for subjects who have taken pairs of antidepressants are depicted as a heat map. Higher pairwise correlations indicate a higher TASI case overlap, suggesting a common underlying mechanism (lower diagonal). The number of participants reporting intake of two antidepressants is depicted on the upper diagonal (minimum *N* = 274)

For each antidepressant taken, participants reported whether or not they had experienced suicidality as a side effect. Given that some individuals had taken more than one antidepressant, several subsets of patients with overlapping TASI data for different antidepressants were available. This overlap enabled us to assess whether TASI occurs independently across different antidepressants. We observed high correlations of TASI across antidepressants (Figure 2), suggesting a common underlying mechanism (which might be explained by a nocebo effect) for TASI across them and a lack of evident greater risk from any particular antidepressant (see discussion).

### 3.3 Clinical risk factors

**FIGURE 3:**
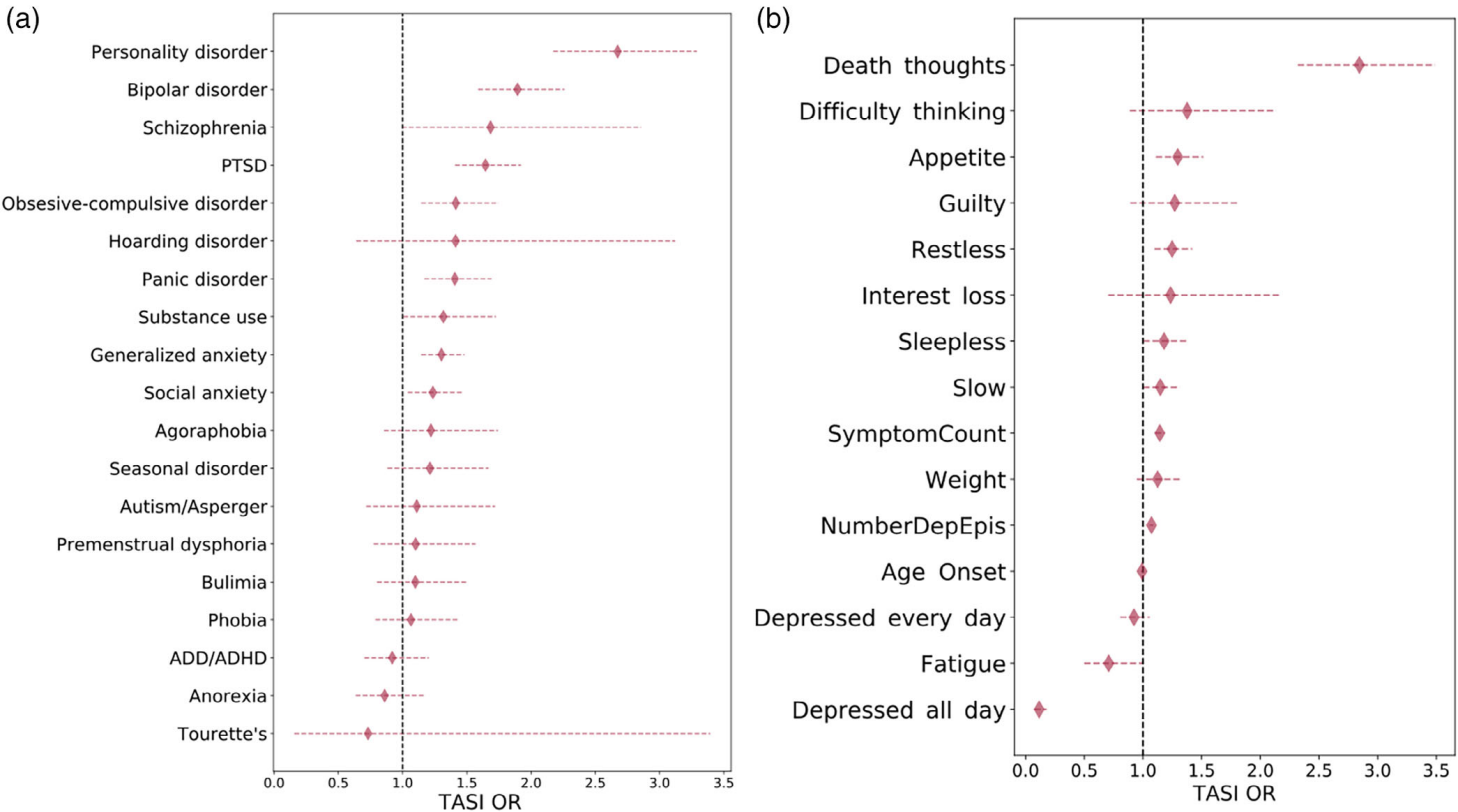
Comorbidities such as bipolar and personality disorder and thoughts of death during depressive episodes are associated with TASI. Forest plots depict the TASI odds ratio for (a) comorbid disorders and (b) depressive symptoms during past depressive episodes. Diamonds represent mean estimates while horizontal lines show 95% CI. ORs were estimated from a multivariate logistic regression accounting for all relevant covariates (see methods)

Six out of 19 comorbidities studied showed an association with TASI in a joint multivariate model (Table S2). Strong positive associations were seen for bipolar post‐traumatic stress and personality disorders. Other comorbidities included obsessive–compulsive disorder, generalized anxiety disorder and panic disorder (Figure 3). Depression severity, approximated by the number of episodes and symptom count, was associated with TASI. Furthermore, depressive symptoms related to TASI included *thoughts of death* (see below and discussion)*, appetite changes*, and *restlessness* (Figure 3). Low‐energy depressive episode characteristics like *fatigue* and *depression lasting all day* were negatively associated with TASI (Figure 3); only the former was statistically significant (Table S3).

### 3.4 TASI genetic risk factors

**FIGURE 4:**
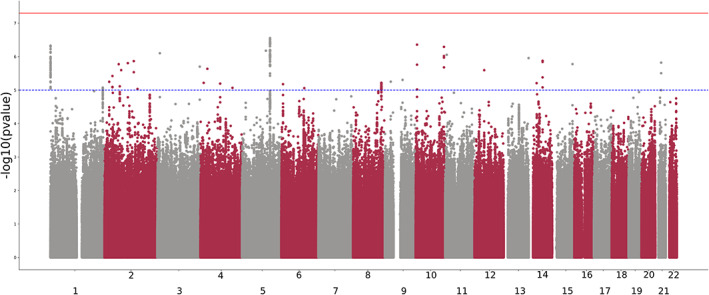
TASI genome‐wide association study. Manhattan plot depicting the results of a genome‐wide association study on TASI. Each dot represents a genetic variant. The position on the y‐axis shows the significance of the association between the variant and TASI. Variants are sorted by chromosome and genomic position. The red (solid) and blue (dashed) lines represent genome‐wide and suggestive significance thresholds, respectively

The GWAS identified no genome‐wide significant hits. Suggestive hits (N = 32; *p* < 1e‐5; independent at R2 0.6) were identified (Figure 4). Overall, no evidence of systematic inflation was identified (Supplementary Figure 2). The LD‐score SNP based heritability was not significant (h2_SNP_ = 0.05, SE = 0.07); this was also true when the SNP based heritability was estimated from GCTA (see methods). Gene‐based tests did not identify any gene associated with TASI beyond genome‐wide significance (*p* < 2 × 10^−6^). Finally, we compared our genome‐wide significant results against a GWAS for treatment increased suicidal ideation in the GENDEP cohort (Perroud et al., 2009) (N ~ 700; 200 cases). Of 27 lead variants with suggestive significance for TASI available in both datasets, only one (rs11966263) showed a nominally significant association (*p* < .05) and the same direction of effect (BETA_AGDS_ = 0.25 SE_AGDS_ = 0.0566; BETA_GENDEP_ = 0.31 SE_GENDEP_ = 0.13) in the replication cohort (Table S4).

### 3.5 TASI prediction

**FIGURE 5:**
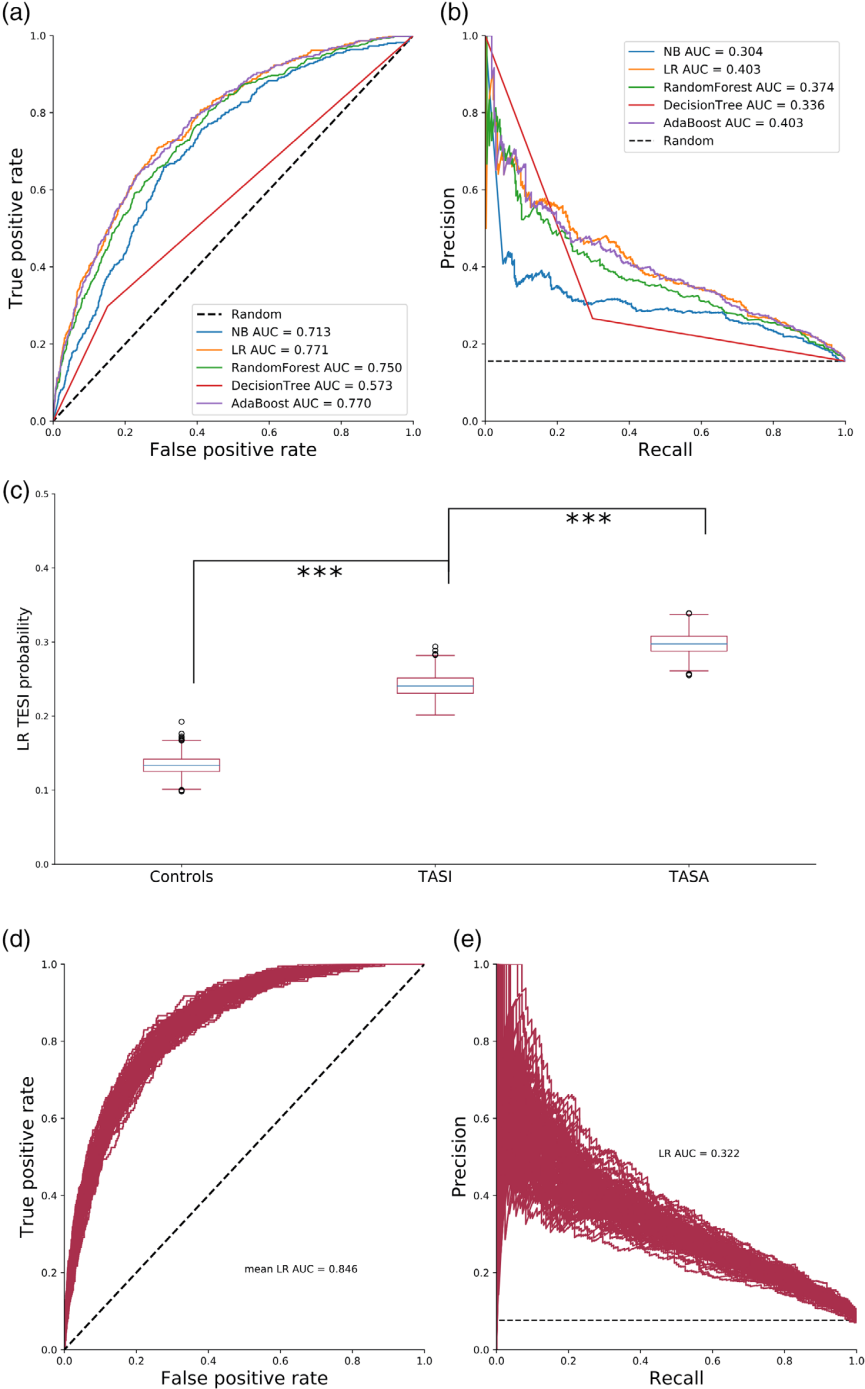
Classifier algorithms trained on TASI can be used to predict TASI and TASA. (a) Receiver operating characteristic (ROC) curve comparing five different machine learning methods for TASI prediction. (b) Curve comparing the precision and recall of the different models. In both graphs, the dashed line represents the expected results from a random guess. Areas under the curve summarize the overall performance of the different models. (c) Distribution of average decision function score (probability) for 1,000 cross‐validations predicting TASI and TASA using the logistic regression model. (d) and (e) Performance of a logistic regression trained to classify TASI used to predict TASA. AUC, area under the curve; LR, logistic regression; NB, Naïve Bayes classifier; (see methods), TASI, treatment attributed suicide ideation; TASA, treatment attributed suicide attempt; Controls, no treatment attributed suicidality. ***p < .001

An ultimate objective of suicide prevention research is to help identify subjects at increased risk for suicide, to inform the design of targeted prevention strategies. Therefore, we built and compared a set of classifiers to test their ability to predict TASI using the risk factors identified in this study. A classifier consists of an algorithm that learns rules from data to output a class prediction (e.g., TASI vs. non‐TASI). We tested the predictive ability of five different classifier algorithms with different underlying assumptions (see Supplementary Methods for details). The best initial models were Logistic Regression (LR) and AdaBoost classifier (AB) (Figure 5a,b). After validation (see Methods), both models showed a consistent and robust behavior as depicted by their ROC curves.

Furthermore, the prediction was not a mere effect of case–control imbalance as shown by the PR curve (Figure S2). We next tested whether some of the strongly associated and potentially circular variables, such as *thoughts of death*, were responsible for the predictive ability of our models. Notably, “*thoughts of death*” was not the only important feature driving prediction. This was also evident for age, which is one of the main risk factors recognized by the FDA for suicidality during antidepressant treatment. After excluding both risk factors as predictors, we observed only a slight decrease in the model's accuracy (Figure S3).

Finally, we tested these models on their ability to stratify subjects given their TASI severity, with the most extreme cases being those who reported TASA (i.e., TASA). Our classifier models could stratify TASA and controls even when they were trained to predict TASI. Furthermore, the three groups (i.e., controls, TASI and TASA) displayed increasing mean TASI probability with increasing suicidality severity (Figure 5c and Figure S4). In fact, classifier models trained to predict TASI showed a high‐prediction accuracy for TASA (Figure 5d,e). To test the external validity of these classifiers, we performed an out of sample prediction of TASA on an independent sample of ~1,600 participants from the Australian Genetics of Bipolar Study. As discussed above, the out of sample prediction showed moderate predictive (AUC = 0.7 and 0.73, Figure S5).

## 4 DISCUSSION

Here, we identified risk factors for TASI and showed that prediction of TASI is possible. Several identified risk factors (e.g., comorbid psychiatric conditions, not being married, depression recurrence) are also known risk factors for suicidality regardless of antidepressant treatment. It is evident that antidepressants are not the main cause of suicide worldwide. In fact, our results suggest that suicidality emerging after treatment for depression could be explained by factors not related to drug class (i.e., individual vulnerabilities or a nocebo effect). Nonetheless, a previous study using structural equation modeling identified evidence for an SNRI specific factor underlying the prevalence of TASI and TASA in the AGDS (Campos et al., 2021) which would imply both general and drug‐class specific factors. In fact, patterns such as an initial increase in energy levels without a decrease in suicidality have been proposed to explain suicidality emerging or increasing following treatment (Licinio & Wong, 2005). Thus, increasing our understanding of the etiology of suicidality and psychiatric illness will be critical to addressing TASI. Several factors such as childhood trauma, anhedonia, sensory processing patterns (e.g., alexithymia), inflammation, glutamatergic, dopaminergic and serotonergic systems have been proposed to drive psychiatric illness outcomes and suicidal behavior (de Berardis et al., 2018; Pompili et al., 2010; Serafini et al., 2016).

Here, we note that a subset of participants self‐report treatment emergence of suicidality and hypothesize that an underlying etiology, probably overlapping the etiology of suicide, might be discoverable. By studying TASI risk factors, we confirm known associations such as younger age (Bridge et al., 2007; Hammad et al., 2006) and discover novel associations, which could be useful for risk stratification. Importantly, our study could not address whether suicidality was, in fact, a side effect of antidepressant treatment (see limitations below) which is still a topic that requires further study.

TASI prevalence in our sample was in‐line with estimates reported by previous studies (Perroud et al., 2012; Rucci et al., 2011). Our results were consistent with an earlier study that identified *melancholic features* and *severe depression* as risk factors for TESI (Zisook et al., 2009). Consistently, we identified symptoms such as *restlessness* and *appetite changes* and higher psychiatric comorbidity associated with a higher risk for TASI. *Thoughts of death* during a depressive episode had a strong association with TASI, even with a more substantial effect than depressive symptom count and the number of previous depressive episodes. This could be interpreted as a potentially circular symptom (i.e., participants referring at the same time to TASI and *thoughts of death*). Nonetheless, this symptom did not fully predict TASI; it was just one of many factors that drove TASI prediction. Psychomotor retardation related symptoms such as *depression lasting all day* were associated with a decreased risk for TASI (with all other symptoms being controlled for), which could be evidence of the symptom remission patterns discussed above (Licinio & Wong, 2005).

In contrast, a previous study (Zisook et al., 2009) failed to detect significant associations of age, marital status, depression recurrence and age of onset with TESI. These features are all known suicidality risk factors (Kposowa, 2000; Lewinsohn, Clarke, Seeley, & Rohde, 1994; Smith, Mercy, & Conn, 1988; Zisook et al., 2007). We interpret this inconsistency as most likely arising from either design differences or a reduced statistical power in the previous study (total *n* = 3,630), which is in line with the observed small effects of some of these variables in our sample.

Randomized controlled studies of emergent suicidality within MDD typically exclude participants with comorbidities, thus disregarding the known co‐occurrence and hierarchy of affective disorders (Laje et al., 2007; Perlis et al., 2007; Perlis, Beasley Jr, et al., 2007; Perroud et al., 2009; Zisook et al., 2009). The concern for TASI arising from a BD misdiagnosis has been raised (Berk & Dodd, 2005; Rihmer & Gonda,  2013). That is consistent with our results suggesting an increase in the odds of developing TASI in subjects who reported having a BD diagnosis. To the best of our knowledge, this is the first study that addresses TASI associations across a range of comorbidities. Future efforts could study personality, bipolar and post‐traumatic stress disorder‐specific symptoms and their potential contribution to TASI.

Our genome‐wide association study identified no significant genome‐wide loci, and a gene‐based test failed to identify any robustly associated genes, thus failing to replicate previous candidate gene associations (Perroud et al., 2009), including *BDNF* (*p* = .087) and *NTRK2* (*p* = .56). The nonsignificant SNP heritability, nonsignificant gene‐based tests, and virtually no suggestive loci replicated in an independent (yet considerably smaller) dataset from the GENDEP cohort (Perroud et al., 2012) suggest that our GWAS analysis is still underpowered. Design differences between the GENDEP study and ours could also explain the lack of replicability. For example, the GENDEP phenotype was treatment increasing suicidal ideation and was assessed longitudinally during a clinical trial. Overall, our genetic analyses would suggest either a lack of genetic basis for TASI (which is not formally tested through the analyses presented here) or that TASI is a complex, highly polygenic trait for which the current sample sizes were underpowered to detect genetic signals. Future studies should focus on boosting sample size and statistical power to robustly unveil the genetic determinants of TASI.

Due to the design and scope of this study, some limitations must be acknowledged. First, ascertainment and data collection were achieved through self‐reported data of a volunteer cohort with participants reporting high levels of educational attainment (a common phenomenon observed in most volunteer‐based recruitment cohorts). We cannot reject the possibility of participant‐specific recall bias. Therefore, we refer to the studied phenotype as treatment attributed suicide ideation instead of the classical term “treatment‐emergent/worsening of suicidal ideation.” Some participants could have misattributed a lack of remission of suicidal ideation due to antidepressant treatment. For that reason, we excluded participants who reported suicidality before initiation of antidepressant therapy.

Furthermore, suicide ideation and attempt definitions could be subjective and heterogeneous across participants. Finally, due to the nature of our study, we could not assess whether TASI is, in fact, a true side‐effect of antidepressant treatment. This would require the existence of an interventional study with a placebo group. We believe studying this phenomenon is relevant, but it is also important to note that recent studies have concluded that antidepressant benefits outweigh suicidality increased risk (Dragioti et al., 2019).

## 5 CONCLUSION

We conducted the largest original study on potential risk factors for TASI to date. TASI prevalence was around 9% in the AGDS, with minor differences across antidepressant types. Our study identified no robust genetic associations with TASI, which suggests it is likely a highly complex and polygenic trait. Our results suggest that being younger, reporting comorbidities such as personality, bipolar and post‐traumatic stress disorders, and characteristic depressive episode symptoms such as having *changes in appetite* are all potential risk factors associated with TASI. Consistent with our original hypothesis, several identified risk factors have been previously linked to suicidality in a context outside antidepressant treatment, suggesting these are largely nondrug‐related factors. However, previous studies linking suicide risk with specific antidepressant classes should be considered. Our classifiers showed some predictive ability even in an independent sample of participants with bipolar disorder. Ultimately, our results could be used to build predictive models that enable clinicians to make informed decisions when prescribing antidepressants and conveying information on possible side effects to their patients, especially those at higher risk, such as younger individuals with psychiatric comorbidities.

## AUTHOR CONTRIBUTIONS

Adrian I. Campos and Miguel E. Rentería conceived, designed the study and wrote the first draft of the manuscript. Adrian I. Campos conducted the analyses with the supervision of Miguel E. Rentería and Nicholas G. Martin and input from Enda M. Byrne, Frank Iorfino, Sarah E. Medland, Naomi R. Wray, and Ian B. Hickie. Chiara Fabbri and Cathryn M. Lewis performed the replication GWAS of the GENDEP study. All co‐authors contributed to the interpretation of the results and provided feedback on the preliminary versions of the manuscript.

## CONFLICT OF INTEREST

Prof Hickie has been: the 2012–2018 commissioner of Australia's National Mental Health Commission; codirector of Health and Policy at the Brain and Mind Centre, University of Sydney; leading community‐based and pharmaceutical industry–supported (Wyeth, Eli Lily, Servier, Pfizer, and AstraZeneca) projects focused on the identification and better management of anxiety and depression; a member of the Medical Advisory Panel for Medibank Private until October 2017; a board member of Psychosis Australia Trust and a member of the Veterans Mental Health Clinical Reference group; and the Chief Scientific Advisor to and an equity shareholder in Innowell. Cathryn Lewis is a member of the Scientific Advisory Board for Myriad Neuroscience. Chiara Fabbri was a speaker for Janssen. Adrian Campos holds equity in Neuren pharmaceuticals. Other authors declared no conflict of interest.

## Supporting information


**Appendix S1** Supporting Information

## Data Availability

Summary statistics for all the analyses performed herein are either available in the supplementary material or via a request to the authors. Raw data cannot be shared due to ethical regulations, but access can be granted by ensuring ethical clearances and approvals. Please contact Nicholas G. Martin at nick.martin@qimrberghofer.edu.au
