# Supplementary material for: Clinical, demographic, and genetic risk factors of treatment‐attributed suicidality in >10,000 Australian adults taking antidepressants
Source: Am J Med Genet B Neuropsychiatr Genet. 2022 Jul 14;189(6):196–206. doi: 10.1002/ajmg.b.32913 (PMC9544797; doi:10.1002/ajmg.b.32913)
Supplement: Supplementary file 1 — Appendix S1 Supporting Information [file AJMG-189-196-s001.docx]

**Supplementary appendix for “Clinical, demographic and genetic risk factors of treatment-attributed suicidality in >20,000 Australian adults on antidepressants”**

Adrian I. Campos,^1,2*^ Enda M. Byrne,^3,4^ Frank Iorfino,^5^ Chiara Fabbri,^6^ Ian B. Hickie,^5^ Cathryn M. Lewis,^6^ Naomi R. Wray,^3,4^ Sarah E. Medland,^1^ Miguel E. Rentería,^1,2*^ Nicholas G. Martin^1^

^1^ QIMR Berghofer Medical Research Institute, Brisbane, QLD, Australia

^2^ Faculty of Medicine, The University of Queensland, Herston, Brisbane, Australia

^3^ Institute for Molecular Bioscience, The University of Queensland, St Lucia, QLD, Australia

^4^ Queensland Brain Institute, The University of Queensland, St Lucia, QLD Australia

^5^ Brain and Mind Centre, University of Sydney, Camperdown, NSW, Australia

^6^ Social, Genetic and Developmental Psychiatry Centre, Institute of Psychiatry, Psychology and Neuroscience, King’s College London, London, UK.

* Correspondence: Adrian I. Campos ([adrian.campos@qimrberghofer.edu.au](mailto:adrian.campos@qimrberghofer.edu.au)) and Miguel E. Rentería ([miguel.renteria@qimrberghofer.edu.au](mailto:miguel.renteria@qimrberghofer.edu.au))

Contents

[Supplementary methods 2](#_Toc34902436)

[Patient recruitment and questionnaires used 2](#_Toc34902437)

[Statistical analyses 2](#_Toc34902438)

[Logistic regression analyses 2](#_Toc34902439)

[Power analyses 2](#_Toc34902440)

[Machine learning methods 2](#_Toc34902441)

[Supplementary Figures 4](#_Toc34902442)

[Supplementary Tables 8](#_Toc34902443)

[Supplementary references 11](#_Toc34902444)

# Supplementary methods

## Patient recruitment and questionnaires used

Recruitment was carried out with assistance from the Australian Government Department of Human Services (DHS). DHS mailed out invitations on behalf of the leading investigators to participate in the Australian Genetics of Depression Study to individuals who had a history of recent antidepressant prescriptions (at least 4 prescriptions in the last 5 years according to information in the Pharmaceutical Benefits Scheme database). Approach letters directed potential participants to an online website ( <https://www.geneticsofdepression.org.au/>), which contained more information about the study and what participation entailed. In parallel, a nation-wide recruitment campaign was conducted using both conventional and electronic social media. The DHS **did not** share any information about the participants invited to participate. **All participants provided informed consent prior to participating in the study. This study and all questionnaires used were approved by the QIMR Berghofer Human Research Ethics Committee.**

## Statistical analyses

Statistical analyses were conducted, and figures were generated using Python. The following modules were used: *pandas, re, numpy, scipy, seaborn, matplotlib, statsmodels* and *scikitlearn* .TASI risk factor ORs were calculated using generalized linear models (GLMs) with a binomial probability distribution and a logit link function using TASI as an outcome variable (logistic regression). Categorical predictors were modelled as matrices of dummy variables. HTML Jupyter Notebooks showing the implementation of the binary classifiers are available upon request.

### Logistic regression analyses

Multivariate effect analyses were performed to estimate the joint effects for all risk factors. TASI was modelled as a binary outcome variable, and all relevant covariates were included as covariates in a single model as exemplified below for antidepressant joint OR calculation:

TASI~sert+escit+venla+amit+mirt+desv+cital+fluox+dulox+parox+Sex+Age+intercept

This estimates the effect (OR) on TASI of intake of each of the antidepressants correcting for the intake of all other antidepressants, thus giving more conservative estimates.

### Power analyses

The power of the present study was assessed by means of a simulation approach. Briefly an artificial dataset with the study sample size was constructed using the same age and sex distributions. A random categorical predictor was added to the model, and a binary outcome variable (simulating TASI) was generated by sampling from a binomial distribution with the probability modelled as a linear combination of effects using a logit function. The coefficients of the artificial predictor variable were varied from -0.5 to 0.25 simulating ORs of ~0.6 to 1.28 respectively. The power of a GLM model to identify a statistically significant association between the artificial predictor and artificial outcome variable was estimated by repeating this approach 1000 times for each coefficient and assessing the percentage of simulations identifying a correct association.

## Supervised learning classifiers

The main objective of these methods is to learn a set of rules to predict an output variable based on a set of input variables or features^1^. In this case the outcome (or random variable) to be predicted was defined to be TASI, and the selected features were: age, sex, marital status, comorbidities, antidepressant consumed and depressive symptoms. Dummy coding was used to include categorical variables such as marital status and comorbidities. To minimise the amount of data loss, missing data (e.g. participants failing to report on their marital status) was coded as an additional category within the dummy coding scheme. Machine learning classifiers were implemented using the *scikit-learn* python module. Because we were interested in testing the predictive capability of the identified variables, we decided to test a variety of different supervised learning algorithms. Below follows a brief description on each of the methods analysed:

**Naïve Bayes**: It is considered a very simple classifier. Technically called a *generative probabilistic classifier* NB tries to estimate (or learn) the joint probabilities: p(feature,outcomes), and later make predictions following Bayes theorem: p(outcomes|features)^2^.

**Logistic Regression**: It is considered a discriminative approach^2^ that *learns* the posterior probability p(outcomes|features). The features are modelled as a linear combination and are linked to the outcome variable through a logit function; it is also a very common statistical analysis tool^3^, but can be used to predict an outcome on an independent dataset. A relevant example where logistic regression is used as a supervised learning classifier is polygenic risk scoring

**Decision Tree**: They are also called classification and regression trees. The training dataset is divided into binary categories based on values of the features. The values that maximize the correct classification (or purity of the classes) determine where to divide the dataset for each feature^1,4^, and prediction is done by following the set of binary rules developed during training. This method is usually combined with parallel (e.g. Random Forest) or ensemble (e.g. AdaBoost) learning approaches.

**Random Forest**: It is defined as a classifier made up of decision tree classifiers^5^. This approach performs a bootstrap sampling of the input sample to train a set of decision trees using only a subset of the features to split the data at each node. The final decision from each tree is averaged to perform the final prediction^1^. Methods of this type are also called *ensemble* modelling, as they rely on building a set or *ensemble* of predictive models to solve the classification problem.

**AdaBoost**: It is also an *ensemble* approach based on combining a set of *weak* (barely better than a random guess) classifiers (also decision trees). A key aspect of the AdaBoost (and other *boosting*) algorithm is that the training data of the weak classifiers is repeated and reweighted at each iteration with a higher weight for misclassified instances^6^.

*Genotyping and imputation*

Samples from the AGDS were genotyped on three different facilities using the same array (GSAMD-24v1-0_20011747). A common set of high QC markers between the different genotyping batches was obtained prior to joint imputation (N=440,664). Marker exclusion criteria (prior to imputation) included: unknown or ambiguous map position and strand alignment in a BLAST search, missingness >5%, p(HWE test)< 10^-6^), MAF<1%, GenTrain score <0.6.  The Michigan imputation server was used to impute the genotypes using the HRCr1.1 as a reference panel. Individuals were excluded based on a high missingness (missing rate > 3%), inconsistent (and unresolvable) sex, or if deemed ancestry outliers from the European population (6 SD deviations from the first two genetic principal component European centroids based on the 1000Genome reference populations).

*GWAS*

GWAS was performed using a subset of unrelated individuals of European ancestry (N= 6,443; cases: 889, controls: 5554) in PLINK1.9. Age, sex, batch and the first 20 genetic principal components were used as covariates. Post GWAS QC included removing variants with a minor allele frequency (MAF) < 1%, and low quality imputation (INFO<0.5). Replication was sought using a previously published GWAS on treatment increased suicidal ideation from the GENDEP study^7^. *LDscore* regression^8^ and *FastBAT* gene-based tests^9^ were performed using the Complex-Traits Genetics Virtual Lab (CTG-VL)^10^.

*GCTA SNP-based heritability estimation*

To estimate SNP-based heritability we employed genomic restricted maximum likelihood (GREML) analysis^11^ as implemented in the tool for Genome-wide Complex Trait Analysis (GCTA) program^12^. Briefly, observed genotype data were used to construct a genetic relatedness matrix (GRM) of the sample. Following Zaitlen *et al* (2013)^13^, a second GRM containing only related individuals was constructed. Both GRMs were used to specify the variance-covariance matrix of two genetic variance components (random effects) on a linear mixed model regression. The first variance component corresponds to the SNP-based heritability while the pedigree based heritability is approximated by the sum of variance explained by both genetic variance components over the total phenotypic variance.

# Supplementary Figures


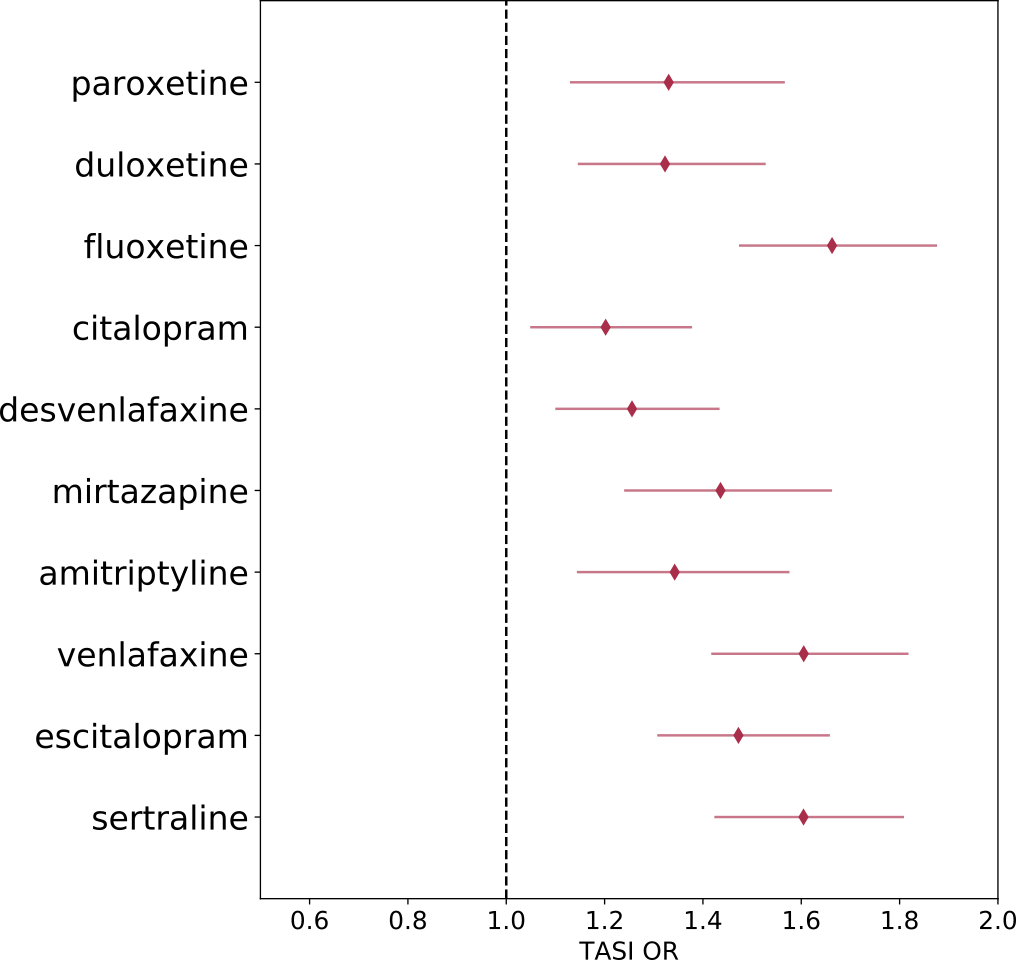


**Supplementary Figure 1. Antidepressant TASI risk**

Antidepressants were used as predictor variables in a multivariate logistic regression to assess their TASI OR. Diamonds represent mean estimates while horizontal lines depict 95% CI.


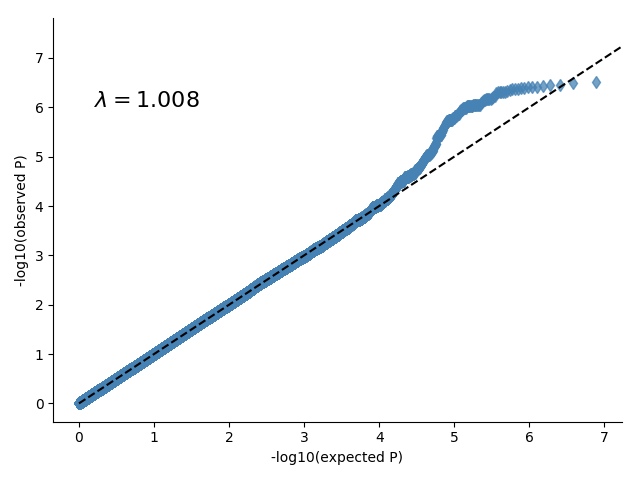


**Supplementary Figure 2 TASI GWAS QQplot**

Quantile-quantile plot showing the comparison between the expected and observed p-values from the GWAS. The global inflation factor is shown within the figure inset.


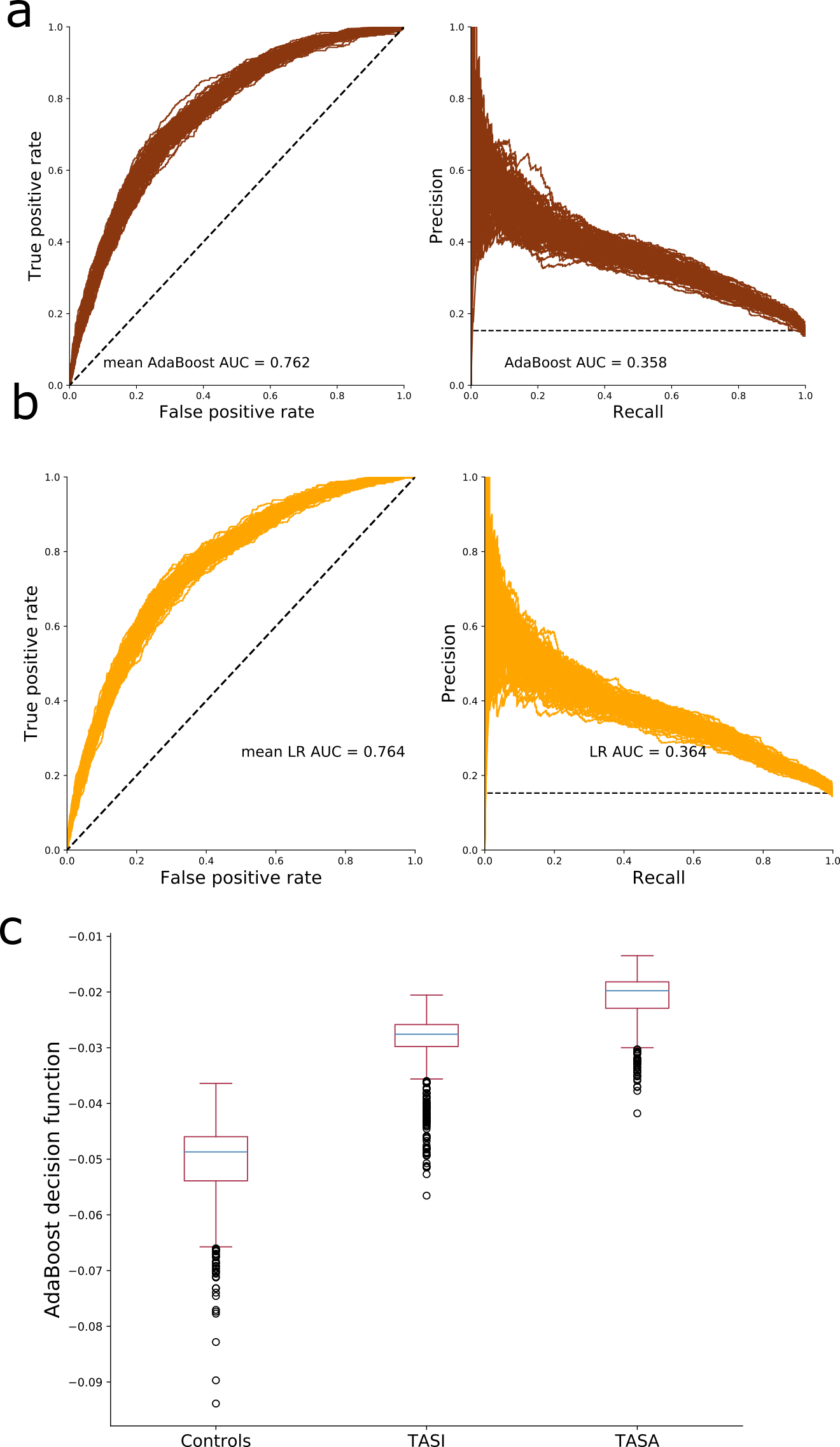


**Supplementary Figure 3 TASI prediction cross-validation**

a-b) 100-fold Cross validation of the best three fitting models logistic regression. c) Depicts the relationship between AB classifier mean decision function and treatment attributed suicidality severity P<0.001


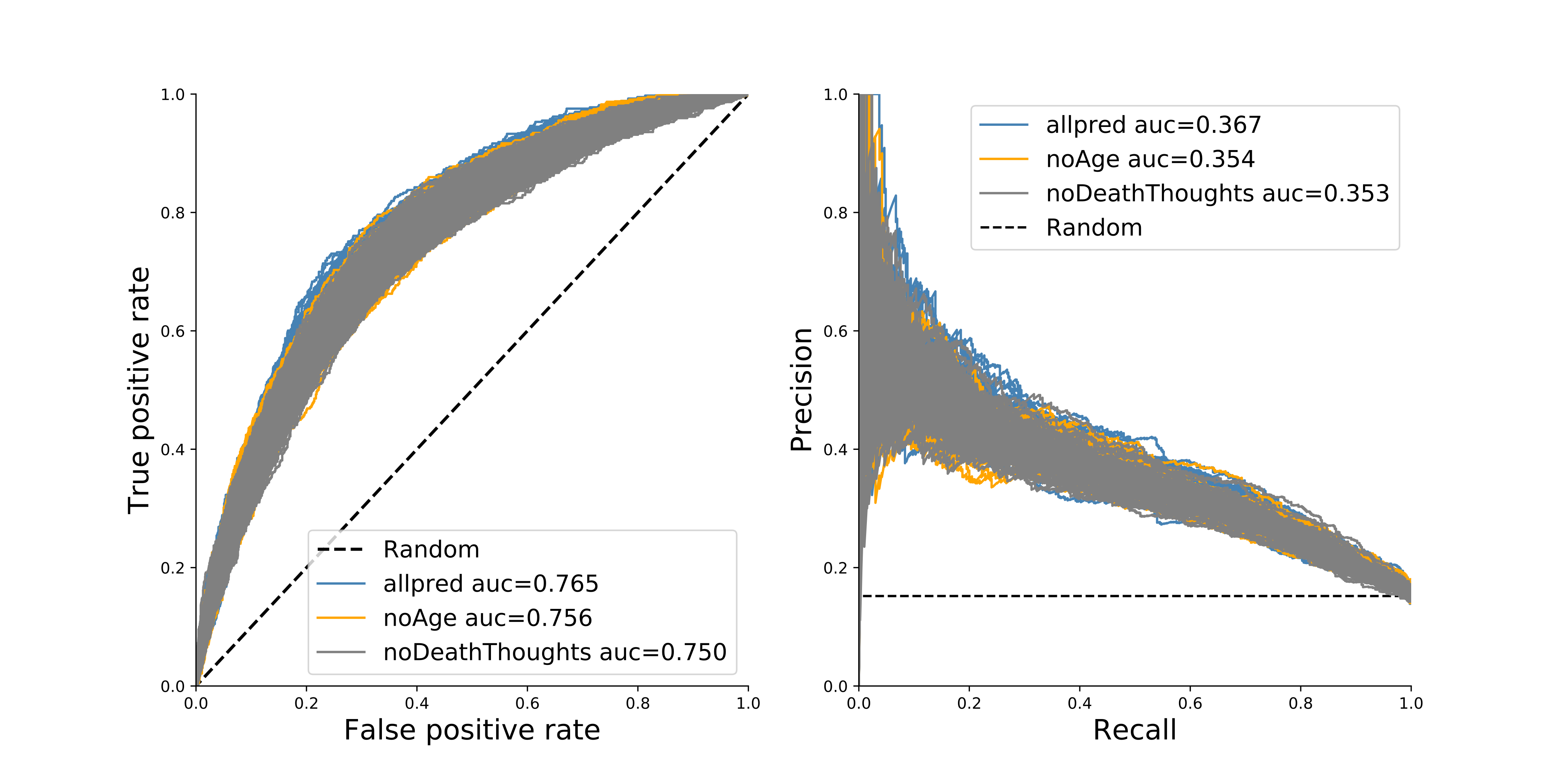


**Supplementary Figure 4 age and thoughts of death are not the only variables driving TASI prediction**

ROC and PR curves profiling the predictive power of logistic regression algorithms trained to predict TASI excluding age and thoughts of death as predictors. One thousand iterations of the training and testing procedure were performed on the full model (allpred), the model excluding age as a predictor (noAge) and the model excluding *thoughts of death* during a depressive episode (noDeathThoughts). The results depict the combined ROC and PR curves, and the mean area under the curve for each category.


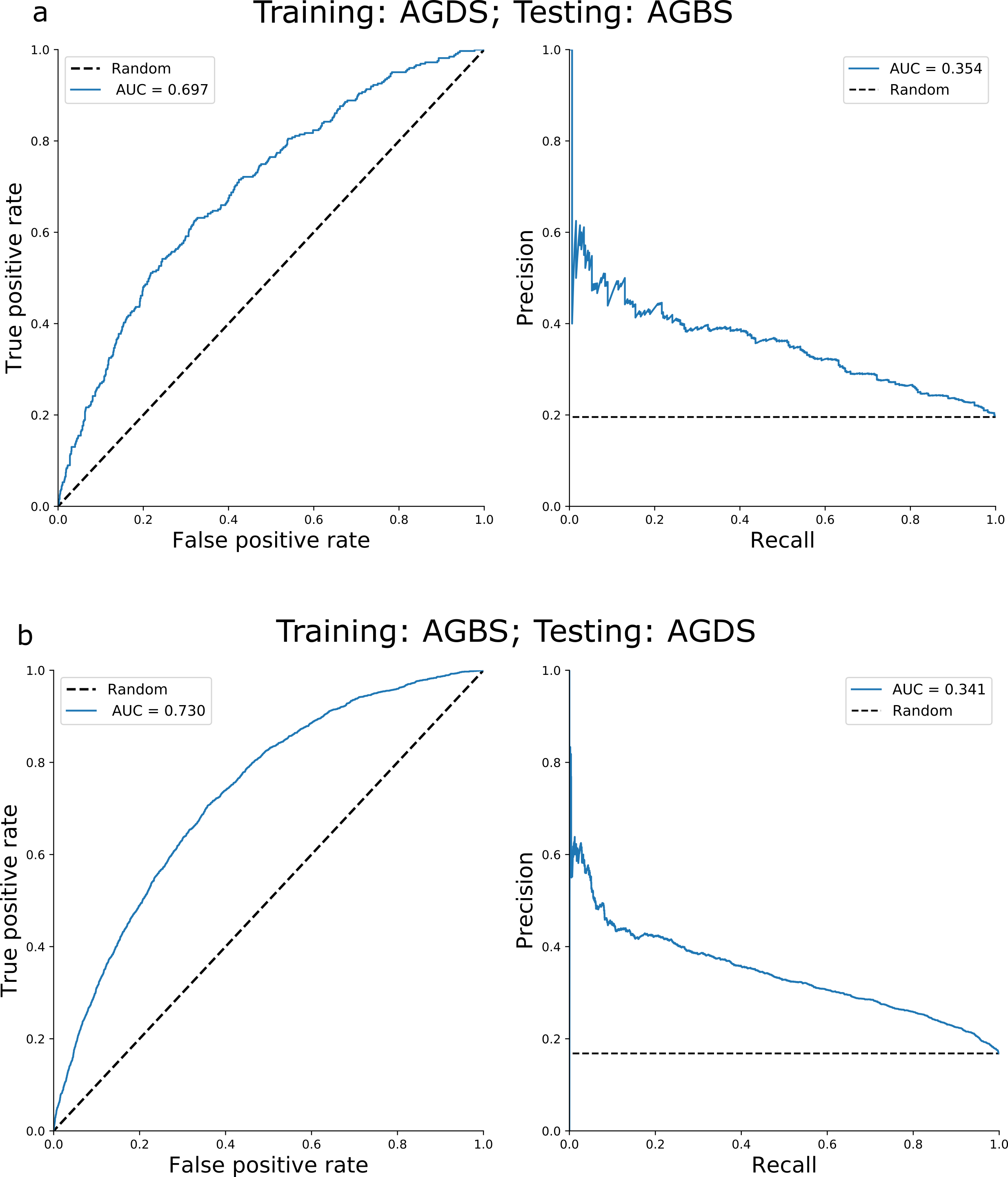


**Supplementary Figure 5 Out of sample prediction**

ROC and PR curves profiling the predictive power of logistic regression algorithms trained to predict TASI in the Australian Genetics of Depression Study (AGDS) and predicting TASI on the Australian Genetics of Bipolar Study. The results depict the ROC and PR curves, and the mean area under the curve.

# Supplementary Tables

| Supplementary table 1. TASI Marital status risk factors | | |
| --- | --- | --- |
| Variable | **OR (95%CI)** | **P-value** |
| Marital status (compared to married) | | |
| Separated | 1.71 | 4.82E-05 |
|  | (1.32-2.21) |  |
| Divorced | 1.94 | 5.69E-11 |
|  | (1.58 -2.36) |  |
| Widowed | 1.46 | 1.47E-01 |
|  | (0.87 -2.47) |  |
| Never Married | 1.25 | 5.29E-03 |
|  | (1.07 -1.47) |  |
| Living with partner | 0.99 | 9.12E-01 |
|  | (0.82 -1.19) |  |

| Supplementary Table 2. comorbidity associations | | | | | | |
| --- | --- | --- | --- | --- | --- | --- |
|  | **OR** | **SE** | **Z** | **P** | **lowCI** | **upCI** |
| Bipolar disorder | 1.894017 | 0.09 | 7.092 | 1.3e-13* | 1.587245 | 2.259176 |
| Premenstrual disphoria | 1.10131 | 0.18 | 0.535 | 0.593 | 0.773368 | 1.568312 |
| Schizophrenia | 1.684384 | 0.269 | 1.935 | 0.053 | 0.993024 | 2.857651 |
| Anorexia | 0.860278 | 0.155 | -0.969 | 0.333 | 0.634448 | 1.166491 |
| Bulimia | 1.099109 | 0.162 | 0.584 | 0.559 | 0.800115 | 1.508325 |
| ADD/ADHD | 0.919339 | 0.138 | -0.611 | 0.541 | 0.701875 | 1.204422 |
| Autism/Asperger | 1.110044 | 0.224 | 0.467 | 0.64 | 0.716054 | 1.721163 |
| Tourette's | 0.731031 | 0.783 | -0.4 | 0.689 | 0.157394 | 3.393969 |
| Generalized anxiety | 1.302128 | 0.066 | 4.014 | 6e-5* | 1.144537 | 1.481418 |
| Panic disorder | 1.406494 | 0.095 | 3.608 | 3e-4* | 1.168826 | 1.69215 |
| Obsesive-compulsive disorder | 1.413827 | 0.108 | 3.205 | 0.001* | 1.144537 | 1.747175 |
| Hoarding disorder | 1.412272 | 0.405 | 0.853 | 0.394 | 0.638905 | 3.120521 |
| PTSD | 1.644769 | 0.079 | 6.272 | 3.5e-10* | 1.40776 | 1.921296 |
| Phobia | 1.06524 | 0.154 | 0.411 | 0.681 | 0.788203 | 1.440514 |
| Seasonal disorder | 1.213125 | 0.163 | 1.186 | 0.236 | 0.881615 | 1.670295 |
| Social anxiety | 1.23553 | 0.088 | 2.404 | 0.016 | 1.03977 | 1.468145 |
| Agoraphobia | 1.220426 | 0.182 | 1.096 | 0.273 | 0.854704 | 1.741941 |
| Personality disorder | 2.673531 | 0.106 | 9.271 | 1.8e-20* | 2.170592 | 3.29037 |
| Substance use | 1.317321 | 0.138 | 1.996 | 0.046 | 1.005013 | 1.726334 |
| *P<0.05 (Bonferroni corrected) | | | | | | |

| Supplementary Table 3 Depressive symptom associations | | | | | | |
| --- | --- | --- | --- | --- | --- | --- |
|  | **OR** | **SE** | **Z** | **P** | **lowCI** | **upCI** |
| Interest_loss | 1.235406 | 0.287 | 0.737 | 0.461 | 0.703984 | 2.166255 |
| Depressed_all_day* | 0.115383 | 0.226 | -9.571 | 1e-21* | 0.074125 | 0.179604 |
| Depressed_every_day | 0.923486 | 0.07 | -1.144 | 0.253 | 0.805735 | 1.058656 |
| Weight | 1.125019 | 0.088 | 1.343 | 0.179 | 0.947432 | 1.336427 |
| Appetite* | 1.296671 | 0.079 | 3.272 | 0.001* | 1.1096 | 1.514371 |
| Sleepless | 1.179865 | 0.083 | 1.994 | 0.046 | 1.003005 | 1.388189 |
| Restless* | 1.248322 | 0.066 | 3.37 | 0.001* | 1.097462 | 1.420487 |
| Slow | 1.147746 | 0.069 | 1.992 | 0.046 | 1.002002 | 1.3139 |
| Fatigue | 0.708362 | 0.178 | -1.935 | 0.053 | 0.499574 | 1.004008 |
| Guilty | 1.270868 | 0.181 | 1.325 | 0.185 | 0.891366 | 1.811219 |
| Difficulty_thinking | 1.376439 | 0.224 | 1.428 | 0.153 | 0.887808 | 2.134004 |
| Death_thoughts* | 2.843683 | 0.104 | 10.031 | 1.12e-23* | 2.318685 | 3.486854 |
| SymptomCount* | 1.143736 | 0.019 | 7.081 | 1.43e-13* | 1.10186 | 1.186491 |
| NumberDepEpis* | 1.072615 | 0.008 | 9.184 | 4.17e-20* | 1.056541 | 1.088717 |
| AgeOnset | 0.991834 | 0.004 | -2.037 | 0.042 | 0.984127 | 1 |
| *P<0.05 (Bonferroni corrected) | | | | | | |

| Supplementary Table 4 GWAS replication results. | | | | | | | | |
| --- | --- | --- | --- | --- | --- | --- | --- | --- |
| Lead SNP | A1agds | A1gendep | BETAagds | BETAgendep | SEagds | SEgendep | Pagds | Pgendep |
| rs118073325 | T | T | 0.370252 | -0.16411 | 0.0833 | 0.185645 | 8.73E-06 | 0.376683 |
| **rs11966263** | **T** | **T** | **0.254952** | **0.312809** | **0.0566** | **0.129197** | **6.65E-06** | **0.015469** |
| rs75996023 | T | T | 0.555321 | 0.266563 | 0.1168 | 0.455197 | 1.99E-06 | 0.558141 |
| rs16890912 | G | G | 0.48403 | -0.53622 | 0.107 | 0.331105 | 6.07E-06 | 0.10534 |
| rs62167308 | C | C | 0.383492 | 0.070766 | 0.0815 | 0.191106 | 2.53E-06 | 0.711161 |
| rs72869202 | C | C | 0.611123 | -0.56046 | 0.1378 | 0.375073 | 9.24E-06 | 0.135108 |
| rs71609571 | T | T | 0.446287 | -0.2804 | 0.0989 | 0.300907 | 6.40E-06 | 0.351419 |
| rs62238229 | G | G | 0.667522 | -0.08281 | 0.1352 | 0.341024 | 7.91E-07 | 0.808137 |
| rs143671886 | A | A | 1.00214 | 0.42449 | 0.2194 | 0.416867 | 4.95E-06 | 0.308539 |
| rs117693660 | T | T | 0.723676 | -0.41686 | 0.1601 | 0.535342 | 6.20E-06 | 0.436167 |
| rs2799058 | G | A | -0.61434 | -0.14542 | 0.1219 | 0.470094 | 4.70E-07 | 0.757064 |
| rs116420697 | A | A | 0.756685 | 0.191595 | 0.1573 | 0.581289 | 1.51E-06 | 0.741703 |
| rs4747880 | T | T | -0.28356 | -0.03494 | 0.0561 | 0.124079 | 4.38E-07 | 0.778267 |
| rs146896146 | T | T | 0.750519 | -0.40828 | 0.1653 | 0.402268 | 5.60E-06 | 0.310127 |
| rs114877845 | G | G | 0.503136 | 0.210601 | 0.1088 | 0.27273 | 3.79E-06 | 0.439996 |
| rs12443264 | G | G | 0.390216 | -0.02103 | 0.0815 | 0.242607 | 1.67E-06 | 0.930912 |
| rs78382527 | G | G | 0.591225 | 0.126888 | 0.1224 | 0.282575 | 1.36E-06 | 0.653412 |
| rs192499138 | G | G | 0.893186 | 0.014731 | 0.186 | 0.492489 | 1.56E-06 | 0.976131 |
| rs3901167 | C | C | 0.376928 | -0.41003 | 0.0846 | 0.246879 | 8.42E-06 | 0.096746 |
| rs71494333 | T | T | 0.436059 | -0.05892 | 0.0868 | 0.228543 | 5.10E-07 | 0.79657 |
| rs4957891 | T | T | 0.293863 | -0.10676 | 0.0591 | 0.124554 | 6.64E-07 | 0.391358 |
| rs72839814 | C | C | 0.290952 | -0.25989 | 0.065 | 0.161401 | 7.71E-06 | 0.107357 |
| rs73225136 | T | T | 0.560701 | -0.26426 | 0.1187 | 0.352614 | 2.31E-06 | 0.453607 |
| rs4665268 | G | A | 0.285404 | -0.04049 | 0.0629 | 0.131896 | 5.64E-06 | 0.75887 |
| rs578111674 | G | G | 0.981067 | -0.088 | 0.2029 | 0.61574 | 1.33E-06 | 0.886354 |
| rs13354462 | T | T | 0.407995 | 0.36481 | 0.0794 | 0.195487 | 2.79E-07 | 0.06202 |
| rs57414252 | C | C | 0.276419 | 0.11947 | 0.0611 | 0.138447 | 6.02E-06 | 0.388173 |

# Supplementary references

1. Crisci C, Ghattas B, Perera G. A review of supervised machine learning algorithms and their applications to ecological data. Ecological Modelling 2012;240:113-22.

2. Ng AY, Jordan MI. On discriminative vs. generative classifiers: A comparison of logistic regression and naive bayes. Advances in neural information processing systems; 2002. p. 841-8.

3. Buschbacher R, Hammond F, Malec J, Nick TG. Handbook for clinical research: design, statistics, and implementation. New York: Demos Medical Publishing; 2014.

4. Speybroeck N. Classification and regression trees. International journal of public health 2012;57:243-6.

5. Breiman L. Random forests. Machine learning 2001;45:5-32.

6. Friedman J, Hastie T, Tibshirani R. Additive logistic regression: a statistical view of boosting (with discussion and a rejoinder by the authors). The annals of statistics 2000;28:337-407.

7. Perroud N, Aitchison KJ, Uher R, et al. Genetic predictors of increase in suicidal ideation during antidepressant treatment in the GENDEP project. Neuropsychopharmacology 2009;34:2517.

8. Bulik-Sullivan BK, Loh P-R, Finucane HK, et al. LD Score regression distinguishes confounding from polygenicity in genome-wide association studies. Nature Genetics 2015;47:291.

9. Bakshi A, Zhu Z, Vinkhuyzen AA, et al. Fast set-based association analysis using summary data from GWAS identifies novel gene loci for human complex traits. Scientific reports 2016;6:32894.

10. Cuellar-Partida G, Lundberg M, Kho PF, D'Urso S, Gutierrez-Mondragon LF, Hwang L-D. Complex-Traits Genetics Virtual Lab: A community-driven web platform for post-GWAS analyses. bioRxiv 2019:518027.

11. Yang J, Benyamin B, McEvoy BP, et al. Common SNPs explain a large proportion of the heritability for human height. Nature genetics 2010;42:565-9.

12. Yang J, Lee SH, Goddard ME, Visscher PM. GCTA: a tool for genome-wide complex trait analysis. The American Journal of Human Genetics 2011;88:76-82.

13. Zaitlen N, Kraft P, Patterson N, et al. Using extended genealogy to estimate components of heritability for 23 quantitative and dichotomous traits. PLoS genetics 2013;9.
